# A systematic review and meta-analysis of cortisol levels in *Plasmodium* infections

**DOI:** 10.1038/s41598-024-68596-0

**Published:** 2024-08-06

**Authors:** Kwuntida Uthaisar Kotepui, Frederick Ramirez Masangkay, Kinley Wangdi, Aongart Mahittikorn, Hideyuki J. Majima, Manas Kotepui

**Affiliations:** 1 Medical Technology Program, Faculty of Science, Nakhon Phanom University, Nakhon Phanom, 48000 Thailand; 2 Department of Medical Technology, Faculty of Pharmacy, University of Santo Tomas, 1008 Manila, Philippines; 3 HEAL Global Research Centre, Health Research Institute, Faculty of Health, University of Canberra, Bruce, ACT 2617 Australia; 4 National Centre for Epidemiology and Population Health, College of Health and Medicine, ANU, Acton, ACT 2601 Australia; 5 Department of Protozoology, Faculty of Tropical Medicine, Mahidol University, Bangkok, 10400 Thailand; 6 Medical Technology, School of Allied Health Sciences, Walailak University, Tha Sala, Nakhon Si Thammarat, 80160 Thailand

**Keywords:** *Plasmodium*, Malaria, Systematic review, Meta-analysis, Cortisol, Severity, Malaria, Diagnostic markers

## Abstract

Malaria has complex interactions with host physiology, including alterations in cortisol levels. Cortisol, a key hormone in the stress response, is known to be dysregulated in various infectious diseases. This systematic review and meta-analysis aimed to elucidate the relationship between *Plasmodium* infection and cortisol levels, shedding light on the intricate interplay between the parasite and the host’s endocrine system. The methodological protocol for assessing cortisol levels in malaria patients was registered in PROSPERO (CRD42024496578), a widely recognized international prospective register of systematic reviews. This registration ensures transparency and minimizes the risk of bias in our research. A comprehensive search strategy was employed across major databases, including Embase, PubMed, Scopus, and Medline, to include studies that reported cortisol levels in infected patients. The qualitative synthesis was undertaken to synthesize the difference in cortisol levels between malaria-infected and uninfected individuals. The meta-analysis employed the random effects model in the quantitative synthesis to calculate the effect estimate. The review included a total of 20 studies, with a substantial number conducted in Africa, followed by Asia and South America. Most included studies (13/20, 65%) reported higher cortisol levels in infected patients than in uninfected patients. The meta-analysis confirmed significantly higher cortisol levels in infected patients compared to uninfected individuals (*P* < 0.0001, standardized mean difference (SMD): 1.354, 95% confidence interval: 0.913 to 1.795, *I*^2^: 88.3%, across 15 studies). Notably, the method for cortisol measurement and the type of blood sample used (serum or plasma) were significant moderators in the analysis, indicating that these factors may influence the observed relationship between *Plasmodium* infection and cortisol levels. The systematic review and meta-analysis confirmed that *Plasmodium* infection is associated with increased cortisol levels, highlighting the intricate relationship between the disease and the host stress response. These findings underscore the potential of cortisol as a supplementary biomarker for understanding the pathophysiological impact of malaria. By providing insights into the stress-related mechanisms of malaria, this comprehensive understanding can inform future research and potentially enhance disease management and treatment strategies, particularly in regions heavily burdened by malaria.

## Introduction

Cortisol, a glucocorticoid hormone produced by the adrenal cortex, serves as a pivotal regulator of various physiological processes essential for maintaining homeostasis^1^. Its secretion is intricately governed by the hypothalamic–pituitary–adrenal (HPA) axis, responding dynamically to stressors encountered by an organism ^2^. Additionally, beyond its role in stress response, cortisol influences immune function^3^ and inflammation^4,5^. Dysregulation of cortisol has been implicated in several diseases, including Cushing syndrome, Addison disease, osteoporosis, hypertension, diabetes mellitus, and the risk of weight gain and subsequent obesity^6–8^. Previous studies have demonstrated a significant association between cortisol levels and the severity of various infections. For instance, elevated cortisol levels at the time of admission have been linked to increased severity of dengue fever^9^. Similarly, increased cortisol levels have been observed in individuals infected with the Influenza B virus (IBV)^10^ and those with Coronavirus disease 2019 (COVID-19)^11^. Furthermore, patients with severe COVID-19 infections have been found to exhibit higher serum cortisol levels^12^.

Malaria, caused by *Plasmodium* parasites transmitted through the bites of infected female *Anopheles* mosquitoes^13^, remains a formidable global health challenge, particularly prevalent in tropical and subtropical regions^14^. The clinical manifestations of malaria range from nonspecific symptoms such as fever, chills, and malaise to severe complications, posing substantial morbidity and mortality burdens worldwide^13^. Despite extensive efforts aimed at malaria control and eradication, the disease continues to exact a heavy toll on vulnerable populations, underscoring the urgency for a deeper understanding of its pathogenesis and associated physiological responses.

Previous studies suggest that malaria-induced immune activation and inflammatory processes may perturb the finely orchestrated regulation of the HPA axis, resulting in alterations in cortisol production, secretion patterns, and responsiveness^15,16^. These alterations, in turn, could modulate the host’s immune competence and susceptibility to severe malaria outcomes^17,18^. Understanding the physiological changes, including alterations in cortisol levels, during *Plasmodium* infections is crucial for several reasons. First, elevated cortisol levels may indicate how the body responds to the stress of infection, potentially influencing disease severity and patient outcomes. Second, cortisol's impact on immune function can affect the host's ability to combat infection, potentially altering susceptibility to severe malaria outcomes. Third, cortisol levels could serve as a biomarker for disease severity, aiding in the timely identification of patients at risk of severe complications. Fourth, understanding the cortisol-malaria relationship could guide the development of targeted treatments that modulate cortisol levels to improve patient outcomes. This systematic review and meta-analysis aimed to investigate the relationship between *Plasmodium* infections and cortisol levels by consolidating existing evidence of alterations in cortisol levels during such infections. The findings from this review have the potential to inform clinical practice, guide future research directions, and enhance strategies for malaria prevention and management globally.

## Methods

### Registration and reporting guideline

The study protocol for assessing cortisol levels in malaria patients was registered in PROSPERO (CRD42024496578), and systematic review and meta-analysis was conducted following the Preferred Reporting Items for Systematic Reviews and Meta-Analyses (PRISMA) guidelines^19^.

### Systematic review question

The systematic review questions followed the Population, Exposure, Comparator, Outcome (PECO) framework^20^. The “P” are patients with suspected malaria; “E” is the *Plasmodium* infection; “C” is *Plasmodium*-uninfected individuals; “O” is blood cortisol levels.

### Definitions

The definition of severe malaria, according to the World Health Organization (WHO), includes clinical or laboratory evidence of vital organ dysfunction. This can manifest as impaired consciousness, prostration, multiple convulsions, severe anemia, respiratory distress in relation to metabolic acidosis, hypoglycemia, renal impairment, jaundice, significant bleeding, shock, and clinical or laboratory evidence of vital organ dysfunction. There are also specific criteria for hyperparasitemia, which differ for *P. falciparum* (> 5% of red blood cells infected) and *P. knowlesi* (hyperparasitemia defined as > 100,000 parasites per µL)^21^. Non-severe malaria is defined as the presence of malaria parasites without evidence of WHO criteria for severe malaria.

### Outcomes

The primary aim of the systematic review and meta-analysis was to synthesize the differences in blood cortisol levels between participants infected with malaria and uninfected individuals. The secondary aim is to compare blood cortisol levels between participants who developed severe malaria and those with less severe forms of the disease. The tertiary aim is to examine the association between blood cortisol levels and parasite density.

### Search strategy

Searches were conducted across major databases, including Embase, PubMed, Scopus, and Medline, using comprehensive terms related to cortisol and malaria. The general search terms were “(Cortisol OR Cortef OR Glucocorticoid OR “Pregn-4-ene-3,20-dione, 11,17,21-trihydroxy-, (11beta)-” OR “Hydrocortisone, (11 alpha)-Isomer” OR Epicortisol OR “11-Epicortisol” OR “11 Epicortisol” OR “Hydrocortisone, (9 beta,10 alpha,11 alpha)-Isomer” OR Cortifair OR Cortril) AND (malaria OR *Plasmodium* OR “*Plasmodium* Infection “ OR “Remittent Fever “ OR “Marsh Fever “ OR Paludism). The search strategy was tailored to each database's specific requirements (Table S1). Additionally, the first 200 results from Google Scholar were reviewed to capture potentially-relevant studies and gray literature, as this method could substantially enhance the clarity and comprehensiveness of systematic reviews^22^. The searches were not limited by language or publication date and were conducted from December 21 to 31, 2023.

### Eligibility criteria

The inclusion criteria for the studies encompassed research conducted in malaria-endemic areas such as Africa, America, and Asia. Eligible study designs included cross-sectional, cohort, case–control, and quasi-experimental studies that reported blood cortisol levels in infected patients. The studies could involve patients with mono or mixed infections of *Plasmodium* species such as *P. falciparum* and *P. vivax*, encompassing adults, children, and pregnant women. Exclusion criteria included animal studies, in vitro experiments, articles without cortisol information, studies measuring cortisol post-treatment or intervention, and repeated participant groups. Non-original articles such as case reports, reviews, and conference abstracts were also excluded to ensure the inclusion of only primary research studies with original data on cortisol levels in malaria patients.

### Study selection and data extraction

After retrieving articles from the main databases, duplicates were removed, and the remaining records were screened using the title and abstract. Articles that met the eligibility criteria were included in the review and underwent full text screening, while those that did not meet the criteria were excluded, with reasons for exclusion specified. Also, the first 200 results from a Google Scholar search were reviewed following a similar process. The study selection was independently performed by two authors (KUK, MK), and disagreements were resolved through discussion with a third author to reach an agreement.

Information was extracted using a standardized datasheet, which included the first author’s name, year of publication and study conduction, study design and location, characteristics, and number of both infected and uninfected participants, exposure characteristics (such as *Plasmodium* species, parasite density, and clinical status) and outcomes of interest (including cortisol levels and qualitative and quantitative data). This also encompassed the methods used for malaria identification and cortisol measurement, including the types of blood samples used. One author (MK) performed data extraction and thoroughly verified by another author (AM).

### Risk of bias assessment

Risk of bias assessment was conducted using the Joanna Briggs Institute (JBI) critical appraisal checklists appropriate to each study design, ensuring a standardized evaluation of methodological quality^23^. The checklist for cross-sectional studies focused on aspects like the clarity of inclusion criteria, detailed descriptions of subjects and settings, validity of exposure and outcome measurements, and handling of confounding factors. The checklist for case–control studies evaluated the comparability of groups, matching of cases and controls, and measurement methods. The cohort study checklist examined the similarity of groups, exposure measurement, identification of confounding factors, and outcome assessment. Lastly, the checklist for quasi-experimental studies assessed the clarity of cause and effect, comparability and treatment of participants, control group presence, outcome measurement methods, and follow-up completeness. Two authors (MK and KUK) performed the risk of bias assessment, and any disagreements were resolved through discussion to reach a consensus.

### Data synthesis and analysis

The qualitative synthesis had been applied to explain the difference in cortisol between malaria-infected patients and uninfected individuals and also between patients with severe and non-severe malaria infection. In the quantitative synthesis, the meta-analysis employed the random effects model to calculate the effect estimate as described previously^24^. The heterogeneity of the included studies was calculated using *I*^2^ statistics (if *I*^2^ is more than 50%, assuming that there is a substantial heterogeneity)^25^. If there was a substantial heterogeneity, the meta-regression and subgroup analyses were conducted to explore potential sources of heterogeneity using covariates extracted from each study such as publication years, study design, continent, participants, *Plasmodium* species, clinical status, method for malaria detection, and method for cortisol measurement. Publication bias was assessed using a funnel plot and Egger’s test^26,27^, which indicated potential bias. An influential analysis (sensitivity analysis) was conducted to determine whether a single study disproportionately affected the overall result^28^. The robustness of the findings was further confirmed through outlier detection, with recalculated results remaining significant even after removing outliers. The power analysis, conducted after the meta-analysis, tested whether the study had sufficient power to detect differences in cortisol levels between comparison groups. This analysis underscored that the sample size and number of included studies were adequate to support the review's conclusions.

## Results

### Search results

**Figure 1:**
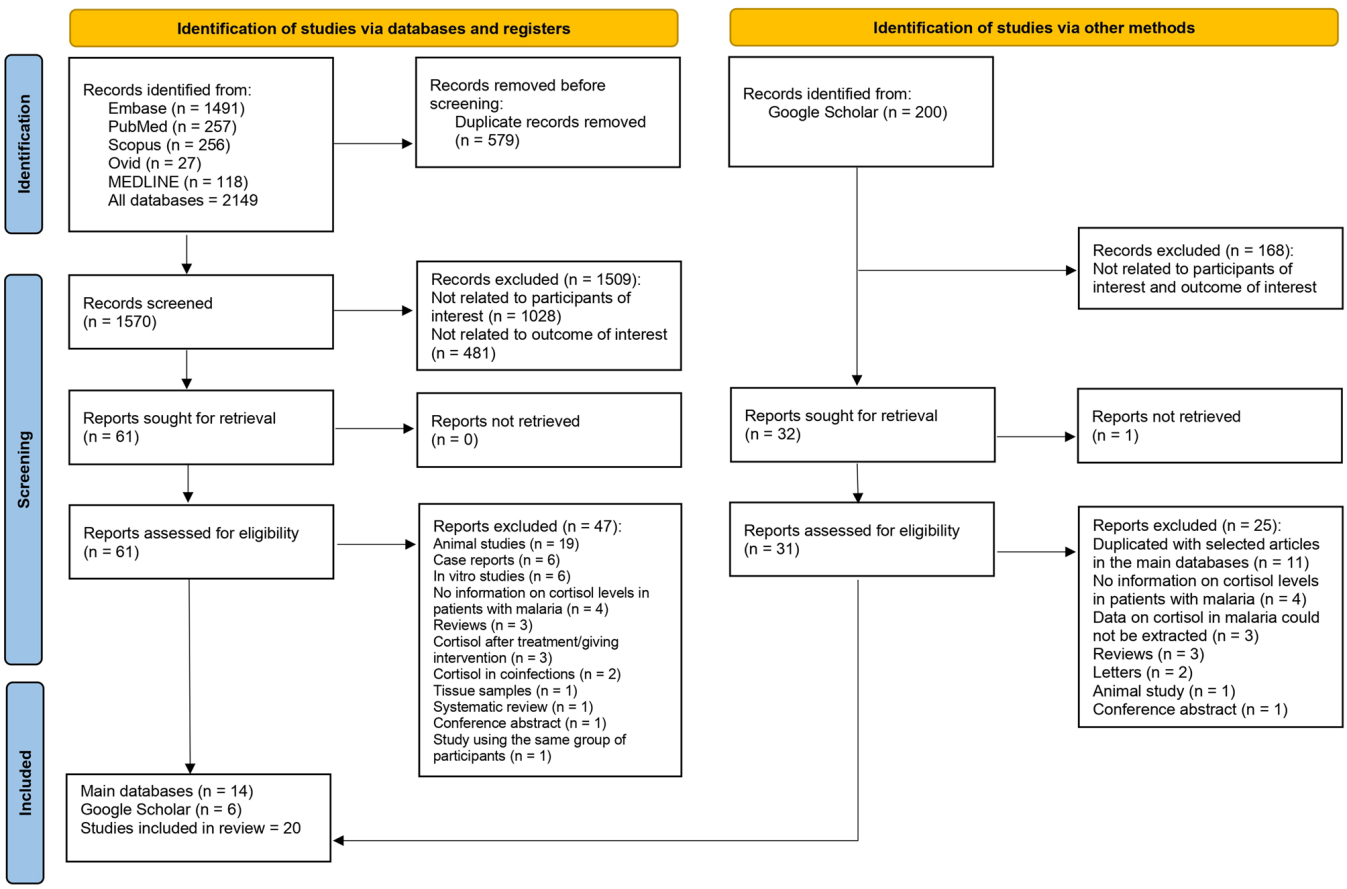
The diagram illustrates the process of selecting studies for inclusion in the systematic review. The left side of the diagram shows the identification and screening process via databases and registers. The right side of the diagram depicts the identification of studies via Google Scholar.

Figure 1 outlines the process of study selection for a systematic review. Initially, 2149 records were identified across various databases, including Embase, PubMed, Scopus, and Medline. After removing 579 duplicates, 1570 records were screened using the title and abstract. Of these, 1509 were excluded for not relating to the participants or outcome of interest. Sixty-one full-text reports were sought for retrieval, with 47 excluded for reasons such as animal studies or case reports with 14 studies shortlisted for the review. The search in Google Scholar with the first 200 results identified 31 relevant reports were assessed for eligibility, with 25 excluded for reasons such as being duplicate records or no information on cortisol levels in malaria. Finally, 20 studies were included in the final review and analysis.

### Characteristics of studies included in the review

Table S2 details the characteristics of studies included in the systematic review. Briefly, the publication years of included studies span from 1987 to 2023, with the majority (40%) published between 2000 and 2009. Study designs varied, with cross-sectional studies (50%) being the most common, followed by case–control (25%), cohort (15%), and quasi-experimental studies (10%). Geographically, these studies were predominantly conducted in Africa (70%), with significant contributions from Nigeria and Sudan. Studies were also from Asian countries like Vietnam, India, Myanmar, and South America (Brazil). The studies primarily focused on *P. falciparum* (80%), with varying participant demographics, including adults, children, and pregnant women. In terms of clinical status, most studies (65%) were from symptomatic malaria patients. Microscopy was the predominant method of *Plasmodium* diagnosis (85%). Cortisol levels are measured using various techniques, with radioimmunoassay (RIA) and enzyme immunoassays (EIA) being the most common. Serum and plasma were the primary blood samples used for cortisol measurement (Table 1).

**Table 1:** Characteristics of included studies.

Characteristics	n (20 studies)	%
Publication years		
2020–2023	3	15.0
2010–2019	4	20.0
2000–2009	8	40.0
Before 2000	5	25.0
Study design		
Cross-sectional studies	10	50.0
Cohort study	3	15.0
Case–control studies	5	25.0
Quasi-experimental	2	10.0
Study area		
Africa	14	70.0
Nigeria	4	20.0
Sudan	4	20.0
Gabon	2	10.0
Cameroon	1	5.0
Ghana	1	5.0
Kenya	1	5.0
Tanzania	1	5.0
Asia	5	25.0
Vietnam	3	15.0
India	1	5.0
Myanmar	1	5.0
South America	1	5.00
Brazil	1	5.0
*Plasmodium* species		
* P. falciparum*	16	80.0
* P. vivax*	1	5.0
Not specified	3	15.0
Participants		
Adults	11	55.0
Children	4	20.0
Pregnant women	4	20.0
Children and adults	1	5.0
Clinical status		
Symptomatic malaria	13	65.0
Asymptomatic malaria	1	5.0
Not defined	6	30.0
Methods for malaria detection		
Microscopy	17	85.0
Microscopy, RDT	3	15.0
Methods for cortisol measurement		
Radioimmunoassay	9	45.0
Enzyme immunoassays	8	40.0
Enzyme linked fluorescent assay	3	15.0
Blood samples for cortisol measurement		
Serum	11	55.0
Plasma	8	40.0
Not specified	1	5.0

RDT, rapid diagnostic test.

### Risk of bias

In the critical appraisal of the included cross-sectional studies using the JBI checklist, most studies^18,29–33^ provided clear inclusion criteria and detailed descriptions of subjects and settings. The validity and reliability of exposure and outcome measurements were generally well-addressed, with only two studies^34,35^ presenting some ambiguities in measurement criteria and outcomes. A notable concern in two studies^35,36^ was the identification and management of confounding factors, which are crucial for establishing causal inferences.

In the critical appraisal of the case–control studies, five studies^37–41^ uniformly ensured comparability between groups and appropriate matching of cases and controls. They all employed consistent criteria for participant identification and uniformly measured exposure. A significant methodological concern was identified across most studies, except for one study, was the lack of identification and strategies to address confounding factors^38^. Additionally, the assessment of whether the exposure period was sufficiently long enough to be meaningful was unclear in several studies. Statistical analyses were appropriate in all studies, and the consistent, reliable outcome assessments were appropriate, except for one study^41^.

In the critical appraisal of the cohort studies, three studies^17,42,43^ ensured that the two groups were similar and recruited from the same population. Exposures were measured consistently, the measurement of exposure was conducted validly and reliably, participants were free of the outcome at the study’s start, and outcomes were measured validly and reliably. Adequate follow-up time, completeness, and management of incomplete follow-up are well-addressed in all studies. Appropriate statistical analyses were utilized across all studies. Two studies^17,42^ also successfully identified confounding factors and stated strategies to deal with them. However, one study^43^ did not identify or articulate strategies for confounding factors.

In the critical appraisal of the quasi-experimental studies, two studies^44,45^ excelled across all the criteria. Both studies clearly defined causal relationships, ensured comparable treatment across groups, included control groups, and conducted multiple outcome measurements. They also managed follow-up processes thoroughly and employed consistent and reliable outcome measurement methods. Appropriate statistical analyses were conducted, affirming the strength of their conclusions (Table S3).

### Qualitative synthesis

The comparison of cortisol levels between malaria cases and uninfected individuals showed that most studies reported higher cortisol levels in infected patients^17,18,30–33,35,37–39,44–46^ (Table 2). Conversely, some studies found no significant difference^29,34,36,40,41,43^. When comparing cortisol levels between severe and non-severe malaria cases, four studies reported significantly higher cortisol levels in severe cases^17,18,33,44^. In contrast, one study found no significant difference between cerebral and uncomplicated malaria^31^. Regarding the relationship between parasitemia and cortisol levels, four studies observed a significant positive correlation^18,39,41,46^, whereas three studies did not find a significant correlation^29,36,42^.

**Table 2:** Blood cortisol levels in infected patients in relation to uninfected individuals, parasite density, and disease severity.

No.	Authors	Study location	*Plasmodium* spp.	Participant	Clinical malaria	Cortisol in malaria patients
1.	Abdagalil et al.^37^	Sudan	*P. falciparum*	Adults aged 20–40 years	Not specified	Blood cortisol levels in infected cases were significantly higher compared to uninfected cases
2.	Adam et al.^34^	Sudan	*P. falciparum*	Pregnant women	Uncomplicated malaria	No significant difference in cortisol levels in patients with malaria and uninfected cases
3.	Armah BNA^29^	Ghana	*P. falciparum*	Children aged 1 to 9 years	Severe and uncomplicated malaria	1. No significant difference in cortisol levels in patients with malaria and uninfected cases 2. No significant correlation was observed between cortisol and parasitemia
4.	Bayoumi et al.^38^	Sudan	*P. falciparum*	Pregnant women	Uncomplicated malaria	Blood cortisol levels in infected cases were significantly higher compared to uninfected cases
5.	Bouyou-Akotet et al.^39^	Gabon	*P. falciparum*	Pregnant women	Asymptomatic malaria	1. Cortisol levels were significantly higher in *P. falciparum*-infected cases than in uninfected cases 2. Cortisol levels were also significantly correlated with the parasite load 3. The *P. falciparum*-infected primigravidae had significantly higher cortisol levels compared to uninfected primigravidae at all time points 4. Infected multigravidae had higher median cortisol levels compared to uninfected multigravidae. 5. Infected primigravidae had higher cortisol levels compared to infected multigravidae
6.	Bouyou-Akotet et al.^46^	Gabon	*P. falciparum*	Pregnant women	Not specified	1. Blood cortisol levels in infected cases were significantly higher compared to uninfected cases 2. Blood concentrations of cortisol in the infected multiparous and primiparous women were significantly higher compared to uninfected controls 3. Cortisol levels were also significantly correlated with the parasite load
7.	Davis et al.^44^	Vietnam	*P. falciparum*	Adults	Severe malaria	Blood cortisol levels in infected cases were significantly higher compared to uninfected cases
8.	Enwonwu et al.^30^	Nigeria	*P. falciparum*	Children aged 14–156 months	Severe and uncomplicated malaria	Blood cortisol levels in infected cases were significantly higher compared to uninfected cases
9.	Ezeugwunne et al.^40^	Nigeria	Not specified	Adults aged 18–60 years	Not specified	No significant difference in cortisol levels in infected cases and uninfected cases
10.	Ibrahim et al.^36^	Sudan	*P. falciparum*	Children and adults	Uncomplicated malaria	1. No significant difference in cortisol levels in infected cases and uninfected cases 2. No correlations between the cortisol levels and parasite density
11.	Libonati et al.^42^	Brazil	*P. falciparum*	Adults aged 15–50 years	Uncomplicated malaria	No significant correlation was observed between cortisol and parasitemia
12.	Olaniyan et al.^41^	Nigeria	*P. falciparum*	Children aged 2–5 years	Not specified	1. No significant difference in cortisol levels in infected cases and uninfected cases 2. There was a significant increase in plasma cortisol with an increase in parasite density
13.	Shwe et al.^31^	Myanmar	Not specified	Adults aged 12–60 years	Severe and uncomplicated malaria	1. Blood cortisol levels in infected cases were significantly higher compared to uninfected cases 2. No significant difference in cortisol levels in patients with cerebral malaria and uncomplicated malaria
14.	Sivaraman et al.^17^	India	*P. vivax*	Adults aged more than 18 years	Severe and uncomplicated malaria	1. Blood cortisol levels in infected cases were significantly higher compared to uninfected cases 2. Blood cortisol levels in patients with severe malaria were significantly higher compared to those with uncomplicated malaria
15.	Ukibe et al.^32^	Nigeria	*P. falciparum*	Adults aged 18 to 40 years	Not specified	Blood cortisol levels in infected cases were significantly higher compared to uninfected cases
16.	van Thien et al.^33^	Vietnam	*P. falciparum*	Adults	Severe malaria	Blood cortisol levels in infected cases were significantly higher compared to uninfected cases
17.	Vandermosten et al.^18^	Cameroon	*P. falciparum*	Children aged 6 months to 17 years	Severe and uncomplicated malaria	1. Blood cortisol levels in infected cases were significantly higher compared to uninfected cases 2. Blood cortisol levels in patients with severe malaria were significantly higher compared to those with uncomplicated malaria 3. Total cortisol levels correlated positively with parasitemia
18.	Vleugels et al.^43^	Kenya	*P. falciparum*	Adults	Not specified	No significant difference in cortisol levels in infected cases and uninfected cases
19.	Vleugels et al.^35^	Tanzania	Not specified	Adults	Not specified	Blood cortisol levels in infected cases were significantly higher compared to uninfected cases
20.	Wilson et al.^45^	Vietnam	*P. falciparum*	Adults aged 21–45 years	Uncomplicated malaria	Blood cortisol levels in infected cases were significantly higher compared to uninfected cases

Regionally, studies from Africa^37,38^ and Asia^33,44^ reported higher cortisol levels in infected adults compared to uninfected cases. In pregnant women, studies from Sudan^34,38^ and Gabon^39^, also found elevated cortisol levels correlated with parasite load. Contrastingly, studies from Ghana^29^ and Nigeria^40^ reported no significant differences. In children, increased cortisol levels in severe malaria were reported in Ghana^29^, Nigeria^30^, Cameroon^18^, and India^17^.

### Meta-analysis

**Figure 2:**
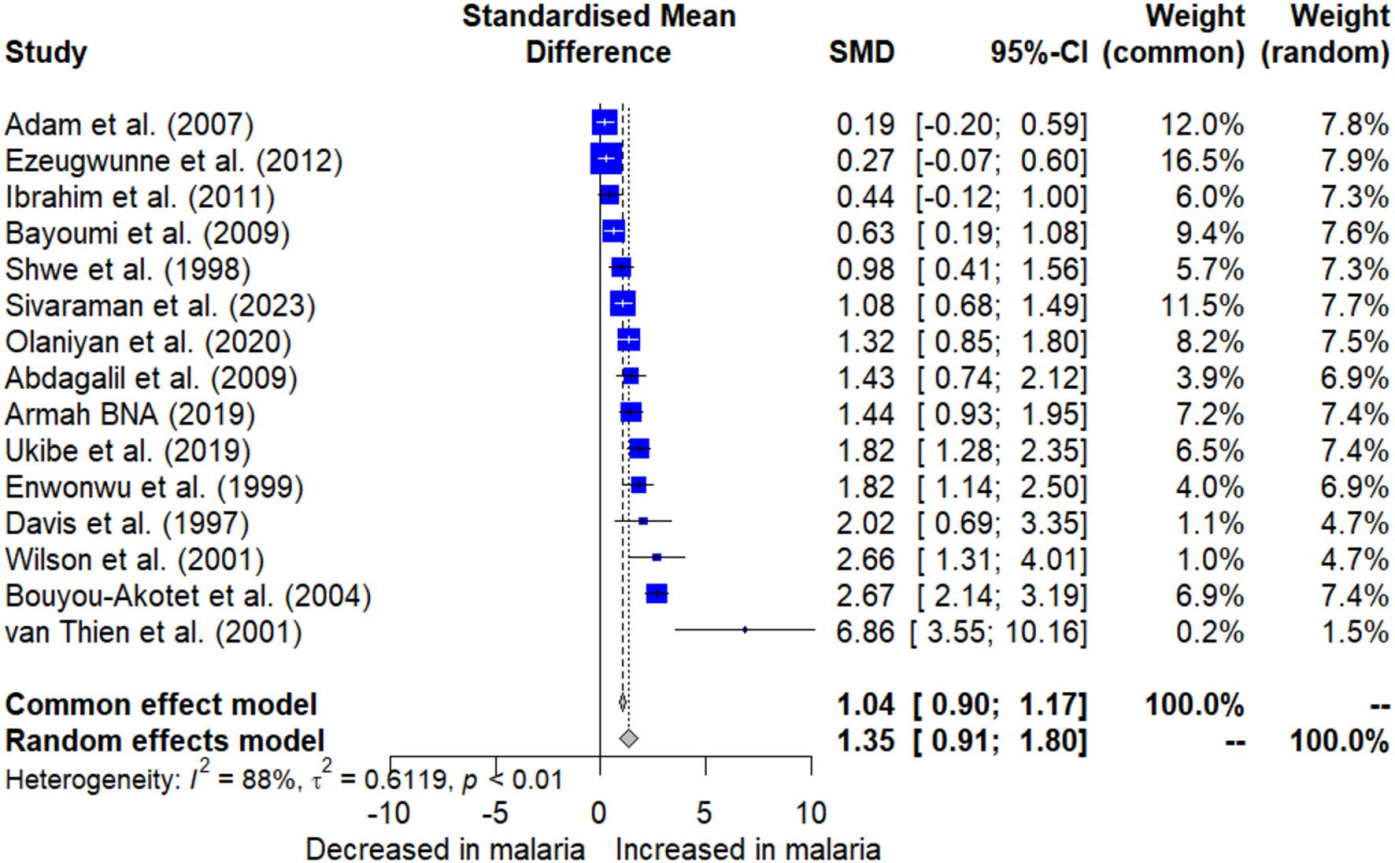
The plot presents the aggregated data on blood cortisol levels from 15 studies, comparing infected patients with uninfected individuals. The Y-axis represents cortisol levels, while the X-axis categorizes the groups compared. The blue square signifies the SMD of cortisol level reported by a study, with error bars depicting the 95% CI. The gray diamond represents the combined SMD with a 95% CI is 1.354 (0.91–1.80), indicating significantly higher cortisol levels in malaria patients (*P* < 0.0001). The high level of heterogeneity among the studies is denoted by an *I*^2^ value of 88.3%. The number of participants across all studies totaled 1053. Abbreviations: SMD, standardized mean difference; CI, confidence interval.

The difference in blood cortisol levels between infected patients and uninfected individuals was estimated using the quantitative data on cortisol as reported by 15 studies^17,29–34,36–38,40,41,44–46^. The results showed significantly higher cortisol levels in infected patients compared to uninfected individuals (*P* < 0.0001, standardized mean difference (SMD): 1.354, 95% CI: 0.913 to 1.795, *I*^2^: 88.3%, across 15 studies, Fig. 2).

The meta-regression analysis of several covariates suggested that none of the covariates—publication years, study design, continent, participants, *Plasmodium* species, clinical status, method for malaria detection, and method for cortisol measurement—significantly explain the heterogeneity (high *I*^2^ values near or above 90% and low R-squared values at 0%), as indicated by the high residual heterogeneity (*P* < 0.0001) and non-significant *P*-values for the test of moderators (ranging from 0.206 to 0.681) for all but two covariates (Table 3). The ‘method for cortisol measurement’ and ‘blood sample for cortisol measurement’ covariates are exceptions. These two covariates demonstrated a significant impact on the between-study variance (with tau^2^ values of 0.415 and 0.387, respectively) and explained a considerable amount of heterogeneity (32.21% and 36.69%, respectively, as indicated by R-squared), with a significant test of moderators *P*-values (0.04 and 0.026, respectively). This suggests that the method by which cortisol is measured, and the type of blood sample used for cortisol measurement are significant moderators in the analysis and account for some of the variability in the effect sizes across studies.

**Table 3:** Meta-regression analysis (15 studies).

Covariates	tau^2^	*I*^2^ (%)	R-squared (%)	Test for residual heterogeneity, *P* value	Test of moderators, *P* value
Publication years	0.660	89.60	0.00	< 0.0001	0.252
Study design	0.635	88.96	0.00	< 0.0001	0.355
Continent	0.678	89.72	0.00	< 0.0001	0.206
Participants	0.799	90.81	0.00	< 0.0001	0.677
*Plasmodium* species	0.648	88.11	0.00	< 0.0001	0.341
Clinical status	0.654	89.26	0.00	< 0.0001	0.681
Method for malaria	0.655	89.71	5.38	< 0.0001	0.639
Method for cortisol measurement	0.415	83.99	32.21	< 0.0001	0.040
Blood sample for cortisol measurement	0.387	82.42	36.69	< 0.0001	0.026

The subgroup analysis of the meta-analysis revealed several insights into how different factors impact cortisol levels in infected patients compared to uninfected individuals (Table 4). The publication year of studies did not significantly affect cortisol differences (*P* = 0.527), suggesting consistency in findings over time. However, the study design played a notable role, with quasi-experimental studies reporting the highest cortisol level differences (*P* = 0.046), indicating that research methodology may influence outcomes. Geographical differences between Africa and Asia did not significantly alter cortisol levels, although the effect was larger in studies from Asia (*P* = 0.194). Notably, participant demographics were influential (*P* = 0.014), with studies on children showing a significant increase in cortisol compared to adults. The differences between *Plasmodium* species (*P. falciparum* or *P. vivax*) did not significantly alter cortisol levels (*P* = 0.095). The methods used for both *Plasmodium* detections did not significantly alter cortisol levels (*P* = 0.324). The methods used for cortisol measurement affect the reported cortisol levels (*P* < 0.0001), with enzyme-linked fluorescent assays yielding the highest differences. Similarly, the type of blood sample used for cortisol measurement was significant (*P* < 0.0001), with plasma samples showing higher cortisol levels compared to serum.

**Table 4:** Subgroup analyses of covariates in the meta-analysis of the differences in cortisol levels between malaria patients and uninfected controls.

Subgroup analyses	Test for subgroup differences (*P* value)	Hedges’ g [95% CI]	*I*^2^ (%)	Number of studies
Publication years	0.527			
2020–2023		1.1827 [0.8742; 1.4913]	0.00	2
2010–2019		0.9788 [0.2357; 1.7218]	90.3	4
2000–2009		1.9766 [0.5824; 3.3708]	93.5	6
Before 2000		1.4938 [0.8220; 2.1656]	53.8	3
Study design	0.046			
Case–control study		0.8711 [0.3203; 1.4218]	82.6	4
Cross-sectional study		1.5567 [0.7712; 2.3421]	91.6	8
Quasi-experimental study		2.3340 [1.3870; 3.2810]	0.00	2
Cohort study		1.0809 [0.6767; 1.4851]	N/A	1
Continent	0.194			
Africa		1.1851 [0.6802; 1.6899]	91.0	10
Asia		2.1833 [0.7658; 3.6008]	77.8	5
Participant	0.014			
Children		1.4709 [1.1602; 1.7816]	0.00	3
Adults		1.5476 [0.8655; 2.2298]	85.6	8
Children and adults		0.4405 [− 0.1211; 1.0021]	N/A	1
Pregnant women		1.1558 [− 0.3306; 2.6422]	96.5	3
*Plasmodium* species	0.095			
* P. falciparum*		1.5459 [1.0087; 2.0832]	88.4	12
* P. vivax*		1.0809 [0.6767; 1.4851]	N/A	1
Not specified		0.5850 [− 0.1120; 1.2820]	77.4	2
Clinical status	0.650			
Symptomatic malaria		1.2663 [0.7262; 1.8064]	82.0	10
Not specified		1.4864 [0.7046; 2.2681]	93.9	5
Methods for *Plasmodium* identification	0.324			
Microscopic method		1.3317 [0.8138; 1.8496]	89.1	13
Microscopic method, RDT		1.6689 [1.2438; 2.0941]	0.00	2
Method for cortisol measurement	< 0.0001			
Radioimmunoassay		0.8732 [0.3885; 1.3579]	78.0	6
Enzyme immunoassays		1.5774 [0.9315; 2.2232]	86.3	8
Enzyme linked fluorescent assay		2.6652 [2.1424; 3.1879]	N/A	1
Blood samples for cortisol measurement	< 0.0001			
Serum		1.0813 [0.5830; 1.5796]	81.6	8
Plasma		1.8795 [1.2959; 2.4630]	80.9	6
Not specified		0.1940 [− 0.2009; 0.5890]	N/A	1

CI, confidence interval; N/A, not assessed.

**Figure 3:**
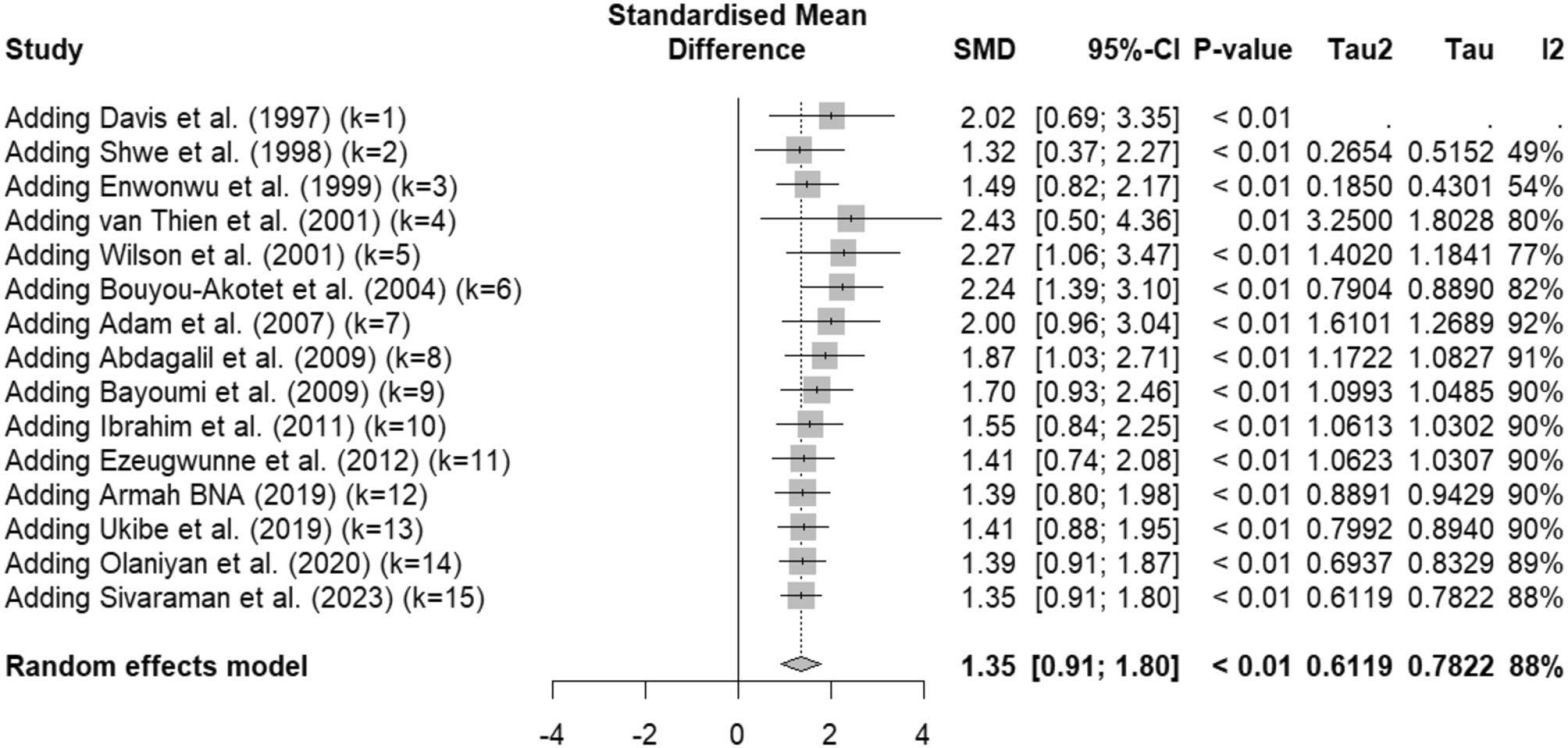
Cumulative analysis of cortisol levels in infected patients versus uninfected individuals. The Y-axis measures cortisol levels, while the X-axis categorizes the studies included in the analysis. Each gray squared box represents data from a single study. The gray diamond represents the combined effect size calculated across all studies. Error bars may indicate confidence intervals, demonstrating the precision of the estimated effect sizes. The heterogeneity of the included studies is quantified by the *I*^2^ statistic. Abbreviations: SMD, standardized mean difference; CI, confidence interval.

A cumulative meta-analysis shows that after each new study is incorporated, the results of the meta-analysis did not significantly change, suggesting that further studies might not substantially alter the conclusion (Fig. 3).

### Publication bias

**Figure 4:**
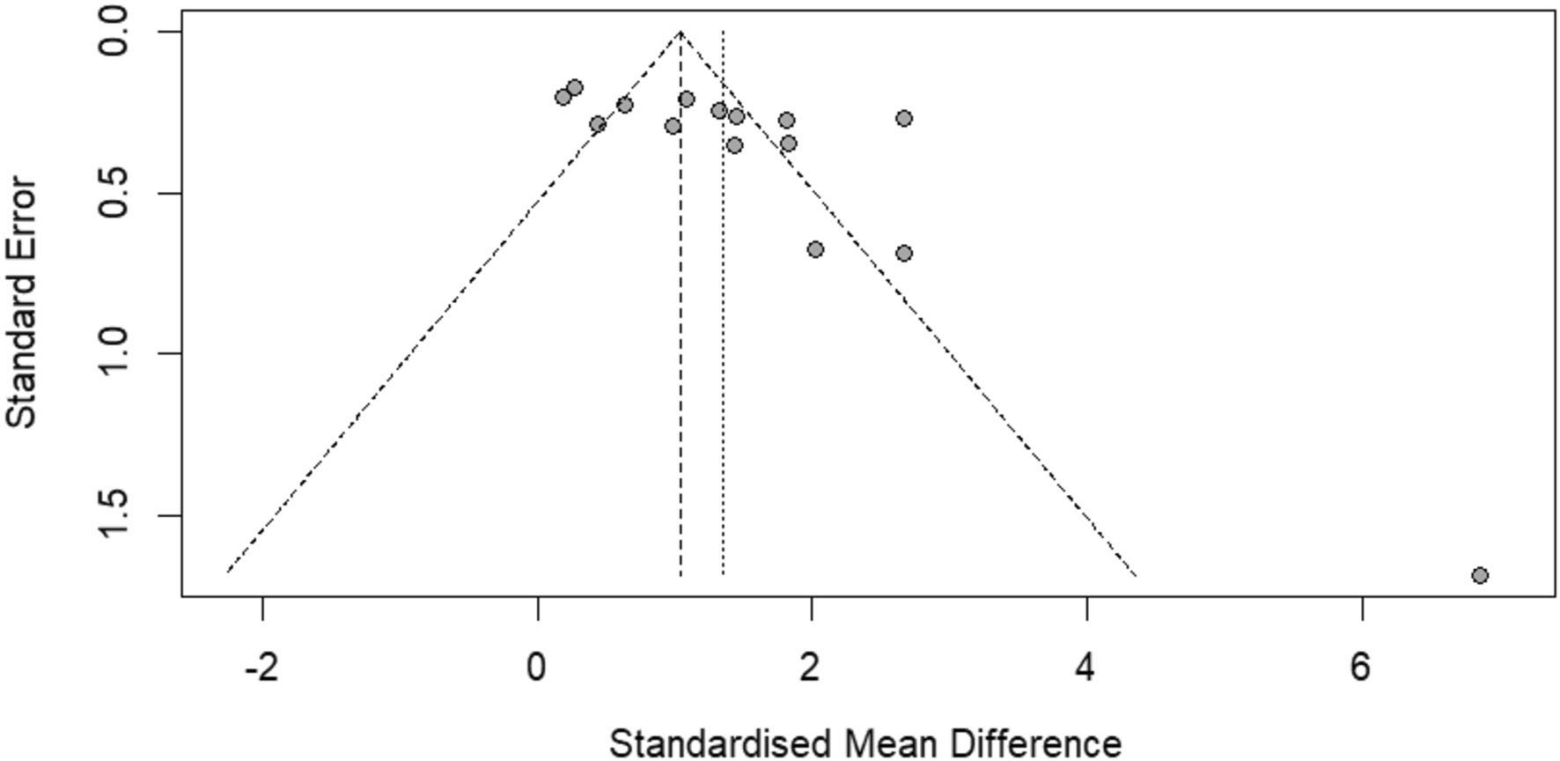
The funnel plot in the context of the meta-analysis compared cortisol levels in infected patients compared to uninfected individuals. Gray dots, effect estimate of a single study.

There is an asymmetry in the funnel plot (Fig. 4); this might suggest the presence of publication bias or other biases, such as methodological differences between smaller and larger studies or true heterogeneity. The Egger’s test confirmed the presence of significant publication bias (*P* = 0.013).

### Influential analysis

The leave-one-out meta-analysis on cortisol levels in infected patients versus uninfected individuals indicated that omitting any single study does not substantially alter the overall conclusion that cortisol levels are significantly higher in infected patients compared to uninfected individuals (Supplementary file 1). This is evidenced by the consistently significant *P*-value of less than 0.0001 across all iterations. The robustness of the pooled SMD suggests that the meta-analysis findings are stable and not overly reliant on any single study.

### Outlier detection

The outlier detection in the meta-analysis, focusing on cortisol levels in infected patients versus uninfected individuals, identifies four studies as outliers^33,34,40,46^ (Supplementary file 2). After removing these outliers, the meta-analysis was recalculated with the remaining 11 studies. The results after removing outliers showed a highly significant *P*-value (< 0.0001), indicating that the effect is robust even after removing outliers.

### Power analysis

The power analysis showed a power of 100%. This analysis was based on a hypothesized effect size (d) of 1.354, with 15 studies included (k = 15) and sample sizes of 589 (n1) and 464 (n2) for the two comparative groups. The significance level was set at 5% (*P* = 0.05), and a high heterogeneity was assumed. A power of 100% indicates that the meta-analysis was highly likely to detect the specified effect size if it truly exists in the population. This high-power level is primarily due to the large cumulative sample size and the number of studies included, providing robust capability to identify the effect.

## Discussion

This systematic review and meta-analysis presented a comprehensive compilation of studies investigating the relationship between *Plasmodium* infection and cortisol levels across different demographics and geographical locations. The systematic review indicated a consistent difference in cortisol levels between infected patients and uninfected cases, with several studies from Sudan, Gabon, Vietnam, Nigeria, India, Cameroon, Tanzania, and Vietnam reporting higher blood cortisol levels in malaria-infected patients compared to uninfected individuals^17,18,30–33,35,37–39,44–46^. Furthermore, the meta-analysis confirms that *Plasmodium*-infected patients exhibit significantly higher cortisol levels compared to uninfected controls, underscoring a robust association between *Plasmodium* infection and elevated cortisol levels. This significant increase in cortisol levels was observed across all age groups, with studies involving adults, pregnant women, and children showing similar results. The random effects model analysis showed a substantial and significant effect but also revealed high heterogeneity between studies. This heterogeneity was explored and explained by several variables included in the model, such as differences in study populations, methods of cortisol measurement, and types of blood samples used (serum or plasma).

Although the majority of included studies showed significantly higher cortisol levels in *Plasmodium* -infected individuals compared to uninfected controls, a few studies (from Sudan, Nigeria, Brazil, and Kenya) reported no significant difference in cortisol levels between infected and uninfected cases^29,34,36,40,41,43^. The discrepancy in the results of studies included in the systematic review suggests a possible variation in cortisol levels between studies. This variation was confirmed by the fact that the studies included in the meta-analysis showed considerable heterogeneity, as indicated by a high *I*^2^ value of 88.3%. Substantial differences in the results of the individual studies could be due to various factors such as study design, participant demographics, geographical areas, methods of cortisol and malaria measurement, or other unidentified variables.

The meta-regression and subgroup analyses revealed that among the various factors assessed, only two covariates were identified as significant moderators including the method for cortisol measurement and the blood sample for cortisol measurement. These covariates not only explained a considerable portion of the heterogeneity (32.2% and 36.7%, respectively) but also significantly impacted the between-study variance, highlighting the importance of the technical aspects of cortisol measurement in interpreting the results. A previous study suggested the immunoassays were reported to be interfered with by cross-reactivity in a complex matrix^47^. Furthermore, different properties of antibodies used in the reaction can cause differences in the specificity of the immunoassays for detecting cortisol levels^48^. The multicenter study revealed that when comparing different mass spectrometry and the immunoassays, there was a high inter-assay variation of true cortisol levels^49^. Mass spectrometry enables precise quantification and significantly minimizes variability among different laboratories^50^. Although the immunoassay is being used in clinical laboratories due to its simplicity and availability, the evidence from the meta-analysis potentially suggested the cautious interpretation of immunoassays for the quantification of cortisol in patients with malaria. It should be compared with a reference method to accurately interpret cortisol levels.

For other covariates, the subgroup analysis demonstrated that the type of study design, particularly quasi-experimental studies, influenced the reported differences in cortisol levels. While the effect was larger in studies from Asia compared to Africa, this difference was not statistically significant, indicating that geographical factors might not be the primary determinant for the observed cortisol level differences. The age of participants was a significant factor, with studies involving children showing a more pronounced increase in cortisol levels compared to studies involving adults. The type of *Plasmodium* species (*P. falciparum* or *P. vivax*) did not significantly affect the observed cortisol level differences, suggesting a general effect of malaria on cortisol levels regardless of the specific species involved. For the variation across participants’ groups, studies focusing on pregnant women, particularly in Gabon, highlighted intricate patterns. For instance, infected primigravidae exhibited higher cortisol levels compared to uninfected primigravidae and infected multigravidae^39^. This evidence might suggest a heightened stress response in first-time pregnant women when infected with malaria. Studies involving children and adults, such as those by Armah BNA^29^ from Ghana and Ibrahim et al.^36^ from Sudan showed varied results, indicating that age may influence the cortisol response to malaria.

For the impact of malaria severity on cortisol levels, some studies indicated that cortisol levels are not only higher in malaria-infected patients but also correlate with the severity of the disease. For instance, Sivaraman et al.^17^ from India and Vandermosten et al.^18^ from Cameroon observed significantly higher cortisol levels in patients with severe malaria compared to those with uncomplicated malaria. Similarly, van Thien et al.^33^ from Vietnam also observed significantly increased cortisol levels in patients with cerebral malaria compared to uninfected groups. The result was mixed for the correlation of cortisol levels with parasite density. Bouyou-Akotet et al.^39^ from Gabon and Olaniyan et al.^41^ from Nigeria found significant correlations, suggesting a possible link between the parasite load and stress response as indicated by cortisol levels. However, other studies, including Armah BNA^29^ from Ghana and Libonati et al.^42^ from Brazil, found no significant correlation. These varied results indicated that the association between parasite density and cortisol levels might be influenced by regional differences in malaria endemicity.

High blood cortisol levels may stimulate gluconeogenesis in humans^51^, suggesting that an increased rate of gluconeogenesis could lead to elevated cortisol levels in patients with malaria. Additionally, cortisol is believed to suppress cell-mediated immunity and may be associated with heightened susceptibility to malaria during pregnancy^39^. This hypothesis is bolstered by in vitro experiments demonstrating that high cortisol levels diminish NK cell cytotoxicity against *P. falciparum*^46^ and downregulate NK cell receptors^52^. High blood cortisol levels in patients with malaria also indicate an intact hypothalamic–pituitary–adrenal (HPA) axis and may be attributed to cytokine release or stress induced by malaria^45^. Libonati et al.^42^ observed that cortisol levels were higher on Day 0 and then declined in tandem with clinical improvement and reduction in parasitemia, suggesting that cytokine production decreases during the convalescence phase of the disease. Furthermore, the elevated cortisol levels in patients with malaria may be attributable to the release of cortisol in response to low blood glucose levels, as hypoglycemia is frequently observed in patients with malaria, particularly in severe cases^53–56^.

Understanding the relationship between cortisol levels and malaria can have implications for managing and treating the disease. For instance, if cortisol levels are indeed higher in severe cases, they could potentially serve as a biomarker for disease severity. The mixed results regarding the correlation between cortisol levels and parasite load warrant further investigation to elucidate the underlying mechanisms. The variation in cortisol response based on demographic factors like pregnancy status and age calls for more targeted studies to understand the physiological underpinnings of these differences. In addition, future research should clarify the potential role of cortisol level measurement in clinical settings, particularly whether it can enhance the management and treatment of malaria or predict severe disease outcomes effectively. The findings suggest that cortisol could serve as a supplementary biomarker for understanding the pathophysiological impact of malaria. However, translating these findings into clinical practice requires further validation through targeted studies. Specifically, research should focus on whether interventions that modulate cortisol levels can positively influence patient outcomes and if cortisol measurement can offer actionable insights beyond current diagnostic and prognostic tools.

While a strong and significant effect was shown by the study results, the high levels of heterogeneity suggested that the results should be interpreted with caution, considering the potential variability of effects across different study settings. This heterogeneity could be attributed to differences in study populations, methods of cortisol measurement, and types of blood samples used (serum or plasma), as demonstrated by the subgroup analyses. It is suggested that future research continue to investigate these sources of variability to improve the consistency and reliability of cortisol level measurement in the context of malaria.

## Conclusion

The systematic review and meta-analysis confirm that *Plasmodium* infection was associated with increased cortisol levels, highlighting the intricate relationship between the disease and the host stress response. These findings underscore the potential of cortisol as a supplementary biomarker for understanding the pathophysiological impact of malaria. By providing insights into the stress-related mechanisms of malaria, this comprehensive understanding can inform future research and potentially enhance disease management and treatment strategies, particularly in regions heavily burdened by malaria.

### Supplementary Information

Supplementary Table S1.

Supplementary Table S2.

Supplementary Table S3.

## Data Availability

All data relating to the present study are available in this manuscript and supplementary files.
