# Supplementary material for: A systematic review and meta-analysis of cortisol levels in Plasmodium infections
Source: Sci Rep. 2024 Aug 6;14:18162. doi: 10.1038/s41598-024-68596-0 (PMC11303744; doi:10.1038/s41598-024-68596-0)
Supplement: Supplementary file 1 — Supplementary Table S1. [file 41598_2024_68596_MOESM1_ESM.docx]

**General keywords**

(Cortisol OR Cortef OR Glucocorticoid OR “Pregn-4-ene-3,20-dione, 11,17,21-trihydroxy-, (11beta)-” OR “Hydrocortisone, (11 alpha)-Isomer” OR Epicortisol OR “11-Epicortisol” OR “11 Epicortisol” OR “Hydrocortisone, (9 beta,10 alpha,11 alpha)-Isomer” OR Cortifair OR Cortril) AND (malaria OR plasmodium OR “Plasmodium Infection“ OR “Remittent Fever“ OR “Marsh Fever“ OR Paludism)

PubMed 21 December 2023

| No. | Key concept | Search terms | Results |
| --- | --- | --- | --- |
| 1. | Cortisol | ("cortisol s"[All Fields] OR "cortisole"[All Fields] OR "hydrocortisone"[MeSH Terms] OR "hydrocortisone"[All Fields] OR "cortisol"[All Fields] OR "cortisols"[All Fields] OR ("hydrocortisone"[MeSH Terms] OR "hydrocortisone"[All Fields] OR "cortef"[All Fields] OR "hydrocortisones"[All Fields]) OR ("glucocorticoids"[Pharmacological Action] OR "glucocorticoids"[MeSH Terms] OR "glucocorticoids"[All Fields] OR "glucocorticoid"[All Fields]) OR ("hydrocortisone"[MeSH Terms] OR "hydrocortisone"[All Fields]) OR ("hydrocortisone"[MeSH Terms] OR "hydrocortisone"[All Fields]) OR ("hydrocortisone"[MeSH Terms] OR "hydrocortisone"[All Fields] OR "epicortisol"[All Fields]) OR "11 epicortisol"[All Fields] OR "11 epicortisol"[All Fields] OR ("hydrocortisone"[MeSH Terms] OR "hydrocortisone"[All Fields]) OR ("hydrocortisone"[MeSH Terms] OR "hydrocortisone"[All Fields]) OR ("hydrocortisone"[MeSH Terms] OR "hydrocortisone"[All Fields] OR "cortril"[All Fields])) | 341,449 |
| 2. | Malaria | ("malaria"[MeSH Terms] OR "malaria"[All Fields] OR "malarias"[All Fields] OR "malaria s"[All Fields] OR "malariae"[All Fields] OR ("plasmodium"[MeSH Terms] OR "plasmodium"[All Fields] OR "plasmodiums"[All Fields] OR "plasmodium s"[All Fields]) OR "Plasmodium Infection"[All Fields] OR "Remittent Fever"[All Fields] OR "Marsh Fever"[All Fields] OR ("malaria"[MeSH Terms] OR "malaria"[All Fields] OR "paludism"[All Fields])) | 124,979 |
| 3. | 1 AND 2 | ("cortisol s"[All Fields] OR "cortisole"[All Fields] OR "hydrocortisone"[MeSH Terms] OR "hydrocortisone"[All Fields] OR "cortisol"[All Fields] OR "cortisols"[All Fields] OR ("hydrocortisone"[MeSH Terms] OR "hydrocortisone"[All Fields] OR "cortef"[All Fields] OR "hydrocortisones"[All Fields]) OR ("glucocorticoids"[Pharmacological Action] OR "glucocorticoids"[MeSH Terms] OR "glucocorticoids"[All Fields] OR "glucocorticoid"[All Fields]) OR ("hydrocortisone"[MeSH Terms] OR "hydrocortisone"[All Fields]) OR ("hydrocortisone"[MeSH Terms] OR "hydrocortisone"[All Fields]) OR ("hydrocortisone"[MeSH Terms] OR "hydrocortisone"[All Fields] OR "epicortisol"[All Fields]) OR "11 epicortisol"[All Fields] OR "11 epicortisol"[All Fields] OR ("hydrocortisone"[MeSH Terms] OR "hydrocortisone"[All Fields]) OR ("hydrocortisone"[MeSH Terms] OR "hydrocortisone"[All Fields]) OR ("hydrocortisone"[MeSH Terms] OR "hydrocortisone"[All Fields] OR "cortril"[All Fields])) AND ("malaria"[MeSH Terms] OR "malaria"[All Fields] OR "malarias"[All Fields] OR "malaria s"[All Fields] OR "malariae"[All Fields] OR ("plasmodium"[MeSH Terms] OR "plasmodium"[All Fields] OR "plasmodiums"[All Fields] OR "plasmodium s"[All Fields]) OR "Plasmodium Infection"[All Fields] OR "Remittent Fever"[All Fields] OR "Marsh Fever"[All Fields] OR ("malaria"[MeSH Terms] OR "malaria"[All Fields] OR "paludism"[All Fields])) | 257 |

Embase 21 December 2023

| No. | Key concept | Search terms | Results |
| --- | --- | --- | --- |
| 1. | Cortisol | 'cortisol'/exp OR cortisol OR 'cortef'/exp OR cortef OR 'glucocorticoid'/exp OR glucocorticoid OR 'pregn-4-ene-3,20-dione, 11,17,21-trihydroxy-, (11beta)-' OR 'hydrocortisone, (11 alpha)-isomer' OR 'epicortisol'/exp OR epicortisol OR '11-epicortisol'/exp OR '11-epicortisol' OR '11 epicortisol'/exp OR '11 epicortisol' OR 'hydrocortisone, (9 beta,10 alpha,11 alpha)-isomer' OR cortifair OR 'cortril'/exp OR cortril | 963,297 |
| 2. | Malaria | malaria:ti,ab,kw,de OR plasmodium:ti,ab,kw,de OR ‘Remittent Fever’:ti,ab,kw,de OR ‘Marsh Fever’:ti,ab,kw,de OR Paludism:ti,ab,kw,de OR malaria/exp | 159,414 |
| 3. | 1 AND 2 | (“blood sedimentation”:ti,ab,kw,de OR “erythrocyte sedimentation”:ti,ab,kw,de OR “erythrocyte sedimentation rate”:ti,ab,kw,de OR “erythrocyte sedimentation rates”:ti,ab,kw,de OR “sedimentation rate”:ti,ab,kw,de OR “sedimentation rates”:ti,ab,kw,de OR “sed rate”:ti,ab,kw,de OR ESR:ti,ab,kw,de OR ESR/exp OR “erythrocyte sedimentation rate”/exp) AND (malaria:ti,ab,kw,de OR plasmodium:ti,ab,kw,de OR ‘Remittent Fever’:ti,ab,kw,de OR ‘Marsh Fever’:ti,ab,kw,de OR Paludism:ti,ab,kw,de OR malaria/exp) | 1,491 |

Scopus 21 December 2023

| No. | Key concept | Search terms | Results |
| --- | --- | --- | --- |
| 1. | Cortisol | TITLE-ABS-KEY ( cortisol OR cortef OR glucocorticoid OR "pregn-4-ene-3,20-dione, 11,17,21-trihydroxy-, (11beta)-" OR "hydrocortisone, (11 alpha)-isomer" OR epicortisol OR "11-epicortisol" OR "11 epicortisol" OR "hydrocortisone, (9 beta,10 alpha,11 alpha)-isomer" OR cortifair OR cortril ) | 253,935 |
| 2. | Malaria | TITLE-ABS-KEY ( malaria OR plasmodium OR "plasmodium infection" OR "remittent fever" OR "marsh fever" OR paludism ) | 160,399 |
| 3. | 1 AND 2 | (TITLE-ABS-KEY ( cortisol OR cortef OR glucocorticoid OR "pregn-4-ene-3,20-dione, 11,17,21-trihydroxy-, (11beta)-" OR "hydrocortisone, (11 alpha)-isomer" OR epicortisol OR "11-epicortisol" OR "11 epicortisol" OR "hydrocortisone, (9 beta,10 alpha,11 alpha)-isomer" OR cortifair OR cortril )) AND (TITLE-ABS-KEY ( malaria OR plasmodium OR "plasmodium infection" OR "remittent fever" OR "marsh fever" OR paludism )) | 256 |

MEDLINE 21 December 2023

| No. | Key concept | Search terms | Results |
| --- | --- | --- | --- |
| 1. | Cortisol AND Malaria | (Cortisol OR Cortef OR Glucocorticoid OR “Pregn-4-ene-3,20-dione, 11,17,21-trihydroxy-, (11beta)-” OR “Hydrocortisone, (11 alpha)-Isomer” OR Epicortisol OR “11-Epicortisol” OR “11 Epicortisol” OR “Hydrocortisone, (9 beta,10 alpha,11 alpha)-Isomer” OR Cortifair OR Cortril) AND (malaria OR plasmodium OR “Plasmodium Infection“ OR “Remittent Fever“ OR “Marsh Fever“ OR Paludism) | 118 |

Ovid 21 December 2023

| No. | Key concept | Search terms | Results |
| --- | --- | --- | --- |
| 1. | Cortisol AND Malaria | (Cortisol OR Cortef OR Glucocorticoid OR "Pregn-4-ene-3,20-dione, 11,17,21-trihydroxy-, (11beta)-" OR "Hydrocortisone, (11 alpha)-Isomer" OR Epicortisol OR "11-Epicortisol" OR "11 Epicortisol" OR "Hydrocortisone, (9 beta,10 alpha,11 alpha)-Isomer" OR Cortifair OR Cortril) AND (malaria OR plasmodium OR "Plasmodium Infection" OR "Remittent Fever" OR "Marsh Fever" OR Paludism) {Including Limited Related Terms} | 27 |

ProQuest 21 December 2023

| No. | Key concept | Search terms | Results |
| --- | --- | --- | --- |
| 1. | Cortisol AND Malaria | (Cortisol OR Cortef OR Glucocorticoid OR "Pregn-4-ene-3,20-dione, 11,17,21-trihydroxy-, (11beta)-" OR "Hydrocortisone, (11 alpha)-Isomer" OR Epicortisol OR "11-Epicortisol" OR "11 Epicortisol" OR "Hydrocortisone, (9 beta,10 alpha,11 alpha)-Isomer" OR Cortifair OR Cortril) AND (malaria OR plasmodium OR "Plasmodium Infection" OR "Remittent Fever" OR "Marsh Fever" OR Paludism) |  |

Google Scholar 22 December 2023

| No. | Key concept | Search terms | Results |
| --- | --- | --- | --- |
| 1. | Cortisol AND Malaria | cortisol AND malaria | The first 200 articles |
